# Rationale and design of a multicenter placebo-controlled double-blind randomized trial to evaluate the effect of empagliflozin on endothelial function: the EMBLEM trial

**DOI:** 10.1186/s12933-017-0532-8

**Published:** 2017-04-12

**Authors:** Atsushi Tanaka, Michio Shimabukuro, Yosuke Okada, Isao Taguchi, Minako Yamaoka-Tojo, Hirofumi Tomiyama, Hiroki Teragawa, Seigo Sugiyama, Hisako Yoshida, Yasunori Sato, Atsushi Kawaguchi, Yumi Ikehara, Noritaka Machii, Tatsuya Maruhashi, Kosuke R. Shima, Toshinari Takamura, Yasushi Matsuzawa, Kazuo Kimura, Masashi Sakuma, Jun-ichi Oyama, Teruo Inoue, Yukihito Higashi, Shinichiro Ueda, Koichi Node, On Behalf of the EMBLEM Trial Investigators

**Affiliations:** 1 Department of Cardiovascular Medicine, Saga University, Saga, Japan; 2 Department of Diabetes, Endocrinology, and Metabolism, Fukushima Medical University, Fukushima, Japan; 3 The First Department of Internal Medicine, School of Medicine, University of Occupational and Environmental Health, Kitakyusyu, Japan; 4 Department of Cardiology, Dokkyo Medical University Koshigaya Hospital, Koshigaya, Japan; 5 Department of Cardiovascular Medicine, Kitasato University Graduate School of Medical Sciences, Sagamihara, Japan; 6 Department of Cardiology, Tokyo Medical University, Tokyo, Japan; 7 Department of Cardiovascular Medicine, JR Hiroshima Hospital, Hiroshima, Japan; 8 Division of Cardiovascular Medicine, Diabetes Care Center, Jinnouchi Hospital, Kumamoto, Japan; 9 Clinical Research Center, Saga University, Saga, Japan; 10 Department of Global Clinical Research, Graduate School of Medicine, Chiba University, Chiba, Japan; 11 Clinical Research and Quality Management Center, University of the Ryukyus Hospital, Nishihara, Japan; 12 Department of Cardiovascular Medicine, Graduate School of Biomedical and Health Sciences, Hiroshima University, Hiroshima, Japan; 13 Department of Endocrinology and Metabolism, Kanazawa University Graduate School of Medical Sciences, Kanazawa, Japan; 14 Division of Cardiology, Yokohama City University Medical Center, Yokohama, Japan; 15 Department of Cardiovascular Medicine, Dokkyo Medical University, Mibu, Japan; 16 Department of Cardiovascular Regeneration and Medicine, Research Institute for Radiation Biology and Medicine, Hiroshima University, Hiroshima, Japan; 17 Department of Clinical Pharmacology and Therapeutics, University of the Ryukyus, Nishihara, Japan

**Keywords:** Empagliflozin, Endothelial function, Reactive hyperemia peripheral arterial tonometry (RH-PAT), Sodium glucose cotransporter 2 (SGLT2) inhibitor, Type 2 diabetes mellitus (T2DM)

## Abstract

**Background:**

Type 2 diabetes mellitus (T2DM) is characterized by systemic metabolic abnormalities and the development of micro- and macrovascular complications, resulting in a shortened life expectancy. A recent cardiovascular (CV) safety trial, the EMPA-REG OUTCOME trial, showed that empagliflozin, a sodium glucose cotransporter 2 (SGLT2) inhibitor, markedly reduced CV death and all-cause mortality and hospitalization for heart failure in patients with T2DM and established CV disease (CVD). SGLT2 inhibitors are known to not only decrease plasma glucose levels, but also favorably modulate a wide range of metabolic and hemodynamic disorders related to CV pathways. Although some experimental studies revealed a beneficial effect of SGLT2 inhibitors on atherosclerosis, there is a paucity of clinical data showing that they can slow the progression of atherosclerosis in patients with T2DM. Therefore, the EMBLEM trial was designed to investigate whether empagliflozin treatment can improve endothelial function, which plays a pivotal role in the pathogenesis of atherosclerosis, in patients with T2DM and established CVD.

**Methods:**

The EMBLEM trial is an ongoing, prospective, multicenter, placebo-controlled double-blind randomized, investigator-initiated clinical trial in Japan. A total of 110 participants with T2DM (HbA1c range 6.0–10.0%) and with established CVD will be randomized (1:1) to receive either empagliflozin 10 mg once daily or a placebo. The primary endpoint of the trial is change in the reactive hyperemia (RH)-peripheral arterial tonometry-derived RH index at 24 weeks from baseline. For comparison of treatment effects between the treatment groups, the baseline-adjusted means and their 95% confidence intervals will be estimated by analysis of covariance adjusted for the following allocation factors: HbA1c (<7.0 or ≥7.0%), age (<65 or ≥65 years), systolic blood pressure (<140 or ≥140 mmHg), and current smoking status (nonsmoker or smoker). Key secondary endpoints include the change from baseline for other vascular-related markers such as arterial stiffness, sympathetic nervous activity, and parameters of cardiac and renal function. Importantly, serious adverse effects independently on the causal relationship to the trial drugs and protocol will be also evaluated throughout the trial period.

**Discussion:**

EMBLEM is the first trial to assess the effect of empagliflozin on endothelial function in patients with T2DM and established CVD. Additionally, mechanisms associating empagliflozin-mediated actions with endothelial function and other CV markers will be evaluated. Thus, the trial is designed to elucidate potential mechanisms by which empagliflozin protects CV systems and improves CV outcomes.

*Trial registration* Unique Trial Number, UMIN000024502 (https://upload.umin.ac.jp/cgi-open-bin/ctr_e/ctr_view.cgi?recptno=R000028197)

**Electronic supplementary material:**

The online version of this article (doi:10.1186/s12933-017-0532-8) contains supplementary material, which is available to authorized users.

## Background

Type 2 diabetes mellitus (T2DM) is characterized by systemic insulin resistance, impaired glycemic homeostasis, increased risk of atherosclerosis, and subsequent cardiovascular (CV) complications [1–3]. The risk of CV disease (CVD) is approximately two to fourfold higher in patients with T2DM than in those without the disease [4, 5]. However, whether glucose-lowering therapy can reduce the CVD risk is controversial [6–8]. The potential effects of agents used to treat T2DM on the CV systems are likely to be independent of their glucose-lowering effect, and they may differ from agent to agent. Therefore, it is vitally important to elucidate the actions, outcomes, and safety of such agents on the CV system. This will enable physicians to appropriately tailor each individual’s glucose-lowering medication to reduce their risk for CV complications and adverse outcomes.

Recent CV safety trials with newer glucose-lowering agents have raised important clinical implications and several research questions [9]. Among them, the EMPA-REG OUTCOME trial—in which the long-term CV safety of empagliflozin was investigated—initially exhibited superior CV benefits relative to placebo treatment in patients with T2DM and established CVD, but later no significant risk reduction in vascular events was noted [10]. Empagliflozin is one of the sodium–glucose cotransporter 2 (SGLT2) inhibitors that work in the proximal renal tubules by diminishing the reabsorption of glucose from the glomerular filtrate back into the circulation. This results in increased urinary glucose excretion and decreased plasma glucose concentrations independent of insulin secretion and insulin action [11, 12]. Although the mechanisms by which empagliflozin could reduce hospitalization for heart failure and mortality remain unclear, several reports have proposed that SGLT2 inhibitors could modulate favorably a wide range of metabolic, neurohormonal, and hemodynamic pathways related to CVD [13–17]. Given the favorable nature of the mechanisms of action of SGLT2 inhibitors which are indeed multifactorial, it is highly conceivable that SGLT2 inhibitors can potentially attenuate atherogenesis and reduce subsequent CV event risks such as myocardial infarction and stroke, despite the vascular event risk profile identified in the EMPA-REG OUTCOME trial [10]. The potential of SGLT2 inhibitors to ameliorate atherosclerosis risk in the clinical setting in the short- and long-term remains to be elucidated.

Impaired endothelial function plays the pivotal role in the pathophysiology of atherogenesis and is usually accompanied by increased oxidative stress and inflammatory responses [18]. In addition to atherosclerosis, impaired endothelial function is associated with various type of CVD, including heart failure [19–22]. Moreover, elevated markers indicative of endothelial dysfunction are closely associated with T2DM [23, 24]. Reactive hyperemia-peripheral arterial tonometry (RH-PAT) is a noninvasive and reproducible examination technique for assessing peripheral microvessel endothelial function [25, 26]. A growing body of evidence suggested that RH-PAT-proven endothelial dysfunction can predict future CV events [27–29], and some drug-intervention studies successfully demonstrated improvements in the endothelial function of patients with T2DM [30–32]. Thus, it seems reasonable to evaluate the beneficial effects of SGLT2 inhibitors on atherosclerosis based on the surrogate marker of endothelial function.

Founded on this rationale, the EMBLEM trial was designed to evaluate the effect of empagliflozin on endothelial function—as assessed by RH-PAT—in patients with T2DM and established CVD. This trial presents an opportunity to further investigate the effects of empagliflozin and to gain a better understanding of the possible mechanisms by which it contributes to mediating endothelial function to elicit beneficial CV effects.

## Methods

### Trial overview and design

The EMBLEM trial is an ongoing, prospective, multicenter, placebo-controlled double-blind randomized, investigator-initiated clinical trial. The trial was designed to test the hypothesis that empagliflozin can improve endothelial function in patients with T2DM and established CVD compared with a placebo. Patients will be recruited and randomized in blinded manner to either empagliflozin or placebo treatment group. Following 24 weeks of treatment, the effects of empagliflozin on endothelial function in comparison to placebo will be evaluated using the RH-PAT index (RHI).

The local institutional review boards and independent ethics committees approved the trial protocol. The trial is to be conducted in full compliance with the articles of the Declaration of Helsinki and according to the Ethical Guidelines for Medical and Health Research Involving Human Subjects established by the Ministry of Health, Labour, and Welfare and the Ministry of Education, Culture, Sports, Science, and Technology in Japan. The EMBLEM trial was registered by the UMIN in October 2016 (ID: 000024502). The trial drugs were purchased from Boehringer Ingelheim.

### Trial population and recruitment

We aim to recruit a total of 110 participants across approximately 17 sites in Japan. Recruitment for the trial began in January, 2017, and will end in October, 2017. Eligible participants for the trial are patients aged ≥20 years with T2DM who meet the enrollment criteria detailed in Table 1. Briefly, eligible patients include those with a diagnosis of T2DM, a HbA1c ranging from 6.0 to 10.0%, on stable glucose-lowering agents for at least 1 month before providing consent, and a previous history of CVD, that includes chronic heart failure (CHF), coronary artery disease (CAD), stroke, occlusive peripheral artery disease, or the presence of coronary artery stenosis (≥50%) as detected by angiography or multi-slice computed tomography. CHF must range from classification I to III in accordance with the New York Heart Association (NYHA) functional classification system. In addition, the NYHA classification and medical treatment for CHF (including angiotensin-converting enzyme inhibitors, angiotensin II receptor blockers, beta-blockers, and diuretics) must remain unchanged for 1 month before consent to participate is provided. Cardiac systolic functions may be reduced or preserved. After initially screening for eligibility using prior medical records, each patient will receive an adequate explanation of the trial plan before they provide written informed consent.

**Table 1:** Comprehensive inclusion and exclusion criteria

Inclusion	Exclusion
Adults (aged ≥20 years)	Type 1 diabetes mellitus
T2DM with HbA1c ≥6.0% and <10.0%, unchanged dosage of glucose-lowering agent(s) within 1 month before consent is provided, investigator considers the patient can start or add/switch to the trial drug	History of diabetic ketoacidosis or diabetic coma within the last 6 months
	Severe renal dysfunction (eGFR < 45 mL/min/1.73 m^2^ or undergoing dialysis)
Patients with at least one of the following conditions:	Serious liver dysfunction (AST or ALT is 3 times higher than site reference value)
CHF (NYHA classification I–III, systolic or diastolic failure) not changed NYHA classification and receiving unchanged heart failure medication within 1 month before consent is provided	
	CHF (NYHA classification IV)
	Hypotension (systolic blood pressure <90 mmHg)
	Pituitary gland dysfunction or adrenal gland dysfunction
History of CAD (myocardial infarction and angina) or cerebral infarction	History of CAD, cerebrovascular disease, or TIA within 3 months before consent
Previous coronary revascularization (PCI and CABG)	History of coronary revascularization (PCI and CABG) within 3 months before consent
Presence of coronary artery stenosis ≥50% luminal narrowing depicted by angiography or MSCT	
	Patients received SGLT2 inhibitor within 1 month before consent
Diagnosis of arteriosclerosis obliterans	
Patients who received an explanation of the study and provided written informed consent	Pregnant or suspected pregnancy
	Lactating
	History of hypersensitivity to ingredients of empagliflozin
	Considered inappropriate for the study by investigators due to other reasons, such as malignant complications

*ALT* alanine aminotransferase, *AST* aspartate aminotransferase, *CABG* coronary artery bypass grafting, *CAD* coronary artery disease, *CHF* chronic heart failure, *eGFR* estimated glomerular filtration rate, *MSCT* multi-slice computed tomography, *NYHA* New York Heart Association, *PCI* percutaneous coronary intervention, *SGLT2* sodium glucose cotransporter 2, *TIA* transient ischemic attack, *T2DM* type 2 diabetes mellitus

### Trial design and follow up

**Fig. 1:**
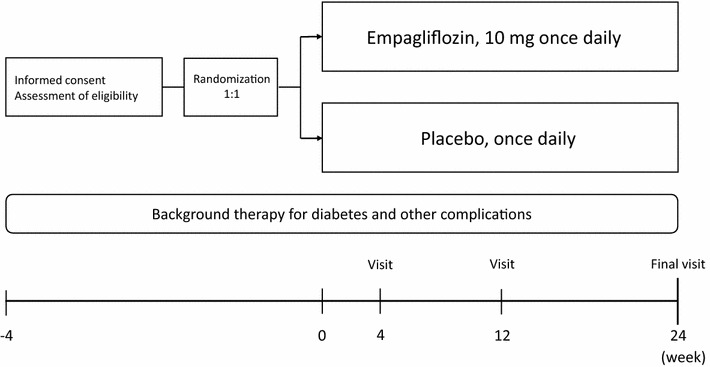
Trial design

All consenting and eligible participants are randomized to receive either empagliflozin or a placebo treatment. Post-randomization follow-up visits are scheduled at 4, 12, and 24 weeks (Fig. 1). Each participant will see their usual-care physician at each visit to receive usual-care and individualized treatment according to their background disease, in addition to administration of the study drug.

### Randomization and blinding

Eligible and consenting participants will be randomized (1:1), in a double-blind manner to either receive empagliflozin (10 mg once daily) or a placebo treatment (once daily), using the web-based minimization dynamic allocation method balancing for HbA1c (<7.0 or ≥7.0%), age (<65 or ≥65 years), systolic blood pressure (BP) (<140 or ≥140 mmHg), and current smoking status (nonsmoker or smoker) at the time of screening [33, 34].

After randomization, patients, investigators, the sponsors, and other individuals involved with various aspects of the trial (e.g., including the conduct or data analysis) are to remain masked to group assignments until after the database is locked and prior to the statistical analysis. Only a predesignated study person will have access to the sealed randomization key codes that will allow unblinding under circumstances considered an emergency and when the identity of the trial drug must be revealed strictly to the investigator for the purpose of providing appropriate medical treatment due to serious adverse effects or ensuring the safety of a trial participant. If the key code for any patient is opened, the principal investigator must be informed immediately of the unblinding incident.

### Treatment

All participants will be followed-up for 24 weeks. Although no specific glycemic target was set (e.g., HbA1c) in the present trial, all participants are to be treated in accordance with the treatment guidelines for T2DM and the official recommendations for appropriate use of SGLT2 inhibitors from the Japan Diabetes Society [35, 36]. Each participant’s pretrial or background glucose-lowering therapy is to remain unchanged during the trial if their medical condition is not compromised by such an approach. However, if participants in either treatment group cannot achieve their glycemic goal, the coadministration of glucose-lowering agents other than SGLT2 inhibitors, or increased dosages of the other glucose-lowering agents will be allowed, if the risk of developing hypoglycemia is minimized. If a patient’s blood glucose exceeds 13.3 mmol/L (>240 mg/dL) after an overnight fast, confirmed by a second measurement on different day, rescue medication can be initiated for the treatment of hyperglycemia. The initiation, choice, and dosage of rescue medication(s) are at the investigator’s discretion. Rescue medication can include an up-titration of a background treatment. If insulin is a background therapy, changes of >10% of the total daily prescribed-dose of insulin are considered rescue therapy. All rescue medication is to be taken in accordance with the prescribing information, with consideration given to minimizing the potential for contraindications and the risk for hypoglycemia. After the trial is completed, all participants can select any glucose-lowering treatments in accordance with their individual requirements.

### Measurements

The baseline characteristics of all patients, including gender, age, body height, body weight (BW), duration of T2DM, complications, background treatment, and smoking and drinking habits are to be recorded prior to randomization. Each participant’s background treatment and trial medication will be recorded at each visit. BP, heart rate (HR), and BW will be measured at baseline and at each visit. The following assessments and methodology will be employed at baseline and at 24 weeks: peripheral endothelial function will be assessed using the RH-PAT test (details described later), the R–R interval coefficient of variation will be determined using electrocardiograms, left ventricular ejection fraction (LVEF) using the modified Simpson method and E/e’ using the tissue Doppler imaging will be measured by echocardiograms, brachial–ankle pulse-wave velocity (baPWV) will be measured using a volume-plethysmographic apparatus as previously described [37]. These physiological tests are to be evaluated locally by technicians at each site. Blood tests will also be performed at baseline and 24 weeks (details listed in Additional file 1), as will specific biomarker assays for N-terminal pro-brain natriuretic peptide (NT-proBNP), interleukin-8 (IL-8), high-sensitivity troponin-I (hs-TnI), receptors for advanced glycation end products (RAGE), and angiopoietin-like protein 2 (ANGPTL2). Biomarkers will be measured at central laboratories. Urinary ketone bodies are to be evaluated qualitatively at each visit. Creatinine-corrected urinary albumin excretion and urinary liver-type fatty acid-binding protein (L-FABP) will be measured at baseline and 24 weeks. In addition, participants will be examined at each visit for symptomatic genital or urinary infections that need to be treated with antibiotics.

#### Measurement of RH-PAT

Peripheral endothelial function will be evaluated by performing RH-PAT using the Endo-PAT2000 device (Itamar Medical, Caesarea, Israel). The principles and measuring procedures of RH-PAT have been described in detail elsewhere [25, 26, 31]. In brief, measurements are performed in the morning at baseline and at 24 weeks, in a quiet, light- and temperature-controlled room, and when the examinee is in a fasted state and in a stable condition before taking their medication for the day. A BP cuff is placed on the upper arm and the opposite arm serves as a control. PAT probes are put on one finger of each hand. After at least a 15-min resting period on a bed in the supine position, the patient’s baseline pulse amplitude is recorded from each fingertip for 6 min. The cuff is subsequently inflated to 60 mmHg above systolic BP or 200 mmHg for 5 min. Finally, the pulse amplitude after cuff deflation is recorded for 5 min. The RHI is automatically calculated by a computerized algorithm in an operator-independent manner [25, 31]. At the same time, the augmentation index (AI) and heart rate variability (HRV), including the standard deviation of the normal to normal intervals (SDNN) and the ratio of low-to high-frequency (LF/HF) are also calculated automatically by using the Endo-PAT2000 software (above version 3.4.4).

### Trial endpoints

The primary endpoint in this trial is the change in RHI from baseline to 24 weeks. Secondary endpoints are baseline to 24 week changes in the following parameters and their correlation with the RHI: double product, calculated as heart rate × systolic BP; baPWV; coefficient of variation of the R–R intervals (including the differences between the results at rest and those during deep breathing) and the standard deviation of heartbeat intervals; LVEF and E/e’; specific biomarkers, including NT-proBNP, IL-8, hs-TnI, RAGEs, and ANGPTL2; renal function, including serum creatinine, eGFR, and creatinine-corrected urinary albumin excretion and L-FABP; glycemic parameters, including HbA1c, fasting blood glucose, and glycoalbumin; clinical parameters, including BP, pulse pressure (systolic BP minus diastolic BP), HR, BW, body mass index, and laboratory measures (details listed in Additional file 1); AI, SDNN, and LF:HF evaluated by the Endo-PAT2000.

### Safety

Throughout the study, safety will be reported for the intention-to-treat population by recording the serious adverse events (AEs) regardless of causal relationship to the trial drugs and protocol, and pre-defined adverse events of special interest (AESI) such as hepatic injury, decreased renal function, metabolic acidosis, ketoacidosis, diabetic ketoacidosis, and events involving lower limb amputation (details in Additional file 2). When the investigators confirm these AEs, their severity or grade, procedures conducted, outcomes, and relationship to the study drug will be assessed. Investigators will promptly report the incidence of AEs to the secretariat who will in turn promptly report to the principal investigator. The principal investigator must report expeditiously to Nippon Boehringer Ingelheim and the Data and Safety Monitoring Board (DSMB). The DSMB consists of an authorized endocrinologist and clinical epidemiologists with relevant expertise. Blinded to treatment allocation, the DSMB will independently evaluate safety during the trial and also assess the necessity for any revisions to the trial design and validate any decisions to continue the trial. If needed, the DSMB will make recommendations on safety issues to the principal investigator. Criteria governing withdrawal from the trial are listed in Table 2.

**Table 2:** Discontinuation criteria

Severe hypoglycemia
Seriously poor glycemic control (HbA1c ≥10.0%)
Development of diabetic ketoacidosis
Serious dehydration requiring rehydration therapy
Considered inappropriate to continue the trial by investigators due to aggravation of primary disease or complications
Considered inappropriate to continue the trial by investigators due to adverse side effects of the trial drug
Considered inappropriate to continue the trial by investigators due to some other reasons
Participant withdrawal of consent

### Statistical considerations

#### Sample size estimation

The possible mode of action of empagliflozin on endothelial function remains to be determined due to the current lack of available evidence. In the EMPA-REG OUTCOME trial, empagliflozin did not affect the incidence of major atherosclerotic macrovascular events such as myocardial infarction and stroke [10], providing results that were comparable to the CV safety trials investigating dipeptidyl peptidase-4 (DPP-4) inhibitors. Empagliflozin markedly reduced the predefined composite CV outcome and hospitalizations for heart failure, whereas the trials assessing DPP-4 inhibitors did not demonstrate such reductions [38–40]. Given these background results, we hypothesized that the putative pharmacological effects of empagliflozin on endothelial function would at least be equivalent to the effect of DPP-4 inhibitors. Therefore, the sample size in the present trial was calculated on the basis of detecting similar treatment effects on endothelial function via assessments of the RHI. Hashikata et al. [32] reported that 3 months of treatment with teneligliptin, a DPP-4 inhibitor, improved the mean (±standard deviation [SD]) RHI from 1.58 ± 0.47 to 2.01 ± 0.72 in patients with T2DM. Therefore, we expected the mean changes in the RHI from baseline would be 0.40 in the empagliflozin group and 0.06 in the placebo group after 24 weeks of treatment with an estimated the difference between groups of 0.34 ± 0.06. At an alpha level of 5% for a two-sided test, a sample size of 50 patients per arm was needed to provide a power of 80% for each comparison. Taking into consideration the possibility of an estimated 10% dropout rate, we estimated that 55 patients per arm (N = 110 patients) will be the appropriate sample size to provide sufficient statistical power for this trial.

#### Statistical analysis plan

The analyses of the primary and secondary endpoints will be performed in the full analysis set, which includes all participants who received at least one dose of treatment during the trial period and who did not have any serious violation of the trial protocol, such as not providing informed consent or registration outside of the trial period.

Summary statistics will be used to calculate the baseline characteristics including the frequencies and proportions for categorical variables and mean ± SD for continuous variables. The patient characteristics will be compared using Chi square tests for categorical variables, t tests will be used for normally distributed continuous variables, and the Wilcoxon rank sum tests will be employed for continuous variables with a skewed distribution.

For the primary analysis—a comparison of treatment effects between the treatment groups—the baseline-adjusted means and their 95% confidence intervals will be estimated by analysis of covariance adjusted for the following allocation factors at the time of screening: HbA1c (<7.0 or ≥7.0%), age (<65 or ≥65 years), systolic BP (<140 or ≥140 mmHg), and current smoking status (nonsmoker or smoker). The primary analysis will not take missing observations into account and the mixed effects model for repeated measures (MMRM) will be used as a sensitivity analysis to examine the effect of missing data. In addition, MMRM will also be used as a sensitivity analysis to assess changes in the RHI during the trial period and be modelled as a function of time and treatment, and the treatment-by-time interaction will be evaluated. Subgroups for the analyses will be defined based on, but not limited to, the RHI at baseline (e.g., <2.0 or <1.67). A secondary analysis will be performed in the same manner as the primary analysis. In addition, correlations between the changes in the RHI and each measurement listed as a secondary endpoint will be evaluated using Pearson’s correlation coefficient.

The principal investigator and a biostatistician will develop the statistical analysis plan before completion of patient recruitment and database lock. All p values will be two-sided, and p values <0.05 will be considered statistically significant. All statistical analyses will be performed using SAS software version 9.4 (SAS Institute, Cary, NC, USA).

### Trial organization and oversight (details in Additional file 3)

The principal investigator of the EMBLEM trial is Koichi Node from the Department of Cardiovascular Medicine at Saga University. The research advisors are Shinichiro Ueda from the Department of Clinical Pharmacology and Therapeutics at the University of the Ryukyus School of Medicine and Yukihito Higashi from the Department of Cardiovascular Regeneration and Medicine at the Research Institute for Radiation Biology and Medicine at Hiroshima University. The steering committee is involved in the planning, operational, analytical, and presentation aspects of the trial. The executive committee will supervise the design and operation of the trial. The roles of the DSMB are described in the section on safety. The trial secretariats are in the Department of Cardiovascular Medicine, Saga University and DOT WORLD CO., LTD., Tokyo, Japan. Data management, monitoring activities, statistical analyses, and audits will be implemented independently on the basis of an outsourcing agreement. The trial drugs, purchased from Boehringer Ingelheim, are to be stored appropriately at the Department of Pharmacy, Saga University Hospital, and will be shipped to each site in response to web-based participant registrations. Data monitoring will be enforced to ensure the research is performed properly, with an independent audit team inspecting several main institutes to ensure the quality of the trial data.

## Discussion

**Fig. 2:**
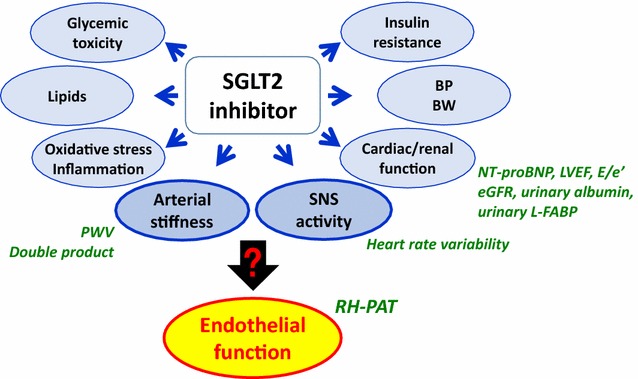
Rationale for the EMBLEM trial. Recent studies have demonstrated that the effects of SGLT2 inhibitors on CV pathways, other than those implicated in glucose-lowering, are multifactorial and possibly result in improved CV outcomes. However, no evidence is currently available to show the effects of SGLT2 inhibitors on endothelial function; *black arrow* shows unestablished pathway, which is a key target in the present trial. Therefore, the EMBLEM trial seeks to assess the effect of empagliflozin on endothelial function through the beneficial effects of SGLT2 inhibitor on other relating CV pathways, such as arterial function and SNS activity, in patients with T2DM and established CV disease. *Blue arrows* show established or expected SGLT2 inhibitor-mediated effects. *BP* blood pressure, *BW* body weight, *CV* cardiovascular, *eGFR* estimated glomerular filtration rate, *L*-*FABP* liver-type fatty acid-binding protein, *LVEF* left ventricular ejection fraction, *NT*-*proBNP* N-terminal pro-brain natriuretic peptide, *PWV* pulse-wave velocity, *RH*-*PAT* reactive hyperemia peripheral arterial tonometry, *SGLT2* sodium–glucose cotransporter 2, *SNS* sympathetic nervous system, *T2DM* type 2 diabetes mellitus

The EMBLEM trial is an ongoing, prospective, multicenter, placebo-controlled, double-blind, randomized, investigator-initiated clinical trial aiming to assess the effect of empagliflozin on endothelial function in patients with T2DM and established CVD. We hypothesized that empagliflozin can improve endothelial function and thereby ameliorate other vascular markers such as arterial stiffness and sympathetic nervous system (SNS) activity in this patient population (Fig. 2). The primary endpoint is the baseline to 24 weeks change in the RH-PAT-derived RHI, a surrogate marker for endothelial function. In addition, other CV markers, such as arterial stiffness and SNS activity, will be also examined as secondary endpoints as will the potential for a mechanistic association between the changes in such markers by empagliflozin treatment. Thus, this trial has the potential to provide novel clinical evidence on the effect of empagliflozin on vascular function and may help to explain the results of the EMPA-REG OUTCOME trial [10].

A growing body of evidence suggests that the effects of SGLT2 inhibitors on CV pathways beyond glucose-lowering actions are multifactorial, possibly resulting in improved CV outcomes [41]. SGLT2 inhibitors are known to not only attenuate glycemic toxicity independent of insulin, but also to reduce BP, BW, and visceral fat via metabolic and hemodynamic pathways [13–16, 42]. These pharmacological effects of SGLT2 inhibitors are likely to contribute to the improvement of beta-cell function and insulin sensitivity [43–45]. Given the potential for these favorable actions, treatment effects of SGLT2 inhibitors on vascular function and atherosclerosis are highly expected. Treatment with SGLT2 inhibitors has already been shown to significantly reduce BP via the modification of markers of arterial stiffness and vascular resistance [46, 47]. In addition, SGLT2 inhibitors can potentially reduce SNS activity that is augmented in part due to hyperglycemia [41, 48], although empagliflozin did not affect SNS activity in younger patients with type 1 diabetes [46]. More recently, some experimental studies showed that SGLT2 treatment could improve circadian rhythm and lability of BP, indicating a possible major cardioprotective role for this class of drugs [49–51]. In the EMPA-REG OUTCOME trial, empagliflozin treatment has elicited marked risk reduction for CV mortality and hospitalization as a result of worsening heart failure in patients with T2DM and established CVD [10]. In contrast, no significant reduction in individual macrovascular events, such as myocardial infarction and stroke, was observed in the EMPA-REG OUTCOME trial or in a subsequent meta-analysis of data from clinical trials [52]. However, because the duration of the trials included in the meta-analysis may be too short to properly evaluate the efficacy of SGLT2 inhibitors in relation to macrovascular events, it may therefore be plausible to investigate the effect of SGLT2 inhibitors on surrogate markers related to vascular function and architecture.

Recent animal studies have demonstrated that treatment with SGLT2 inhibitors can reduce oxidative stress and the inflammation processes that are closely associated with and essential to the genesis of endothelial dysfunction and atherosclerosis [53–55]. Some recent preclinical animal studies provide evidence of SGLT2 inhibitor-mediated mitigation of endothelial dysfunction and/or atherosclerosis. Salim et al. [56] reported that ipragliflozin prevented the development of endothelial dysfunction, partly via the attenuation of oxidative stress in streptozotocin-induced diabetic mice. Nakajima et al. [57] found that ipragliflozin treatment prevented progression of aortic atherosclerosis and improved the survival rate in Apo E-deficient mice. Terasaki et al. [58] also demonstrated that dapagliflozin or ipragliflozin treatment reduced aortic atherosclerotic lesions, accompanied by suppression of macrophage foam cell formation, in diabetic Apo E-deficient and db/db mice. More recently, Han et al. [59] revealed that empagliflozin-mediated attenuation of aortic atheroma volume in Apo E-deficient mice was associated with reduced insulin resistance, a reduction in the levels of inflammatory markers, and increased adiponectin. These results provide strong evidence to suggest the possibility that SGLT2 inhibitor effects are potent enough to exert vascular protective effects in clinical settings; however, little evidence currently exists to show the clinical effects of SGLT2 inhibitors on vascular function and atherosclerosis in patients with T2DM. Some randomized clinical trials designed to assess the anti-atherosclerotic effects of SGLT2 inhibitors by measuring carotid intima-media thickness are currently underway [60, 61].

In the present trial, we focused on vascular endothelial function which has a pivotal role in the regulation of vascular tone and circulation which itself is governed by the interplay of vasoconstrictive and vasodilative factors. Endothelial function can be impaired by various metabolic disturbances and is closely associated with increased oxidative stress, inflammation, and insulin resistance [62, 63]. Hence, endothelial dysfunction is paramount to the pathogenesis of atherosclerosis and is considered to be an important marker predictive of the future risk of CV events [64–66]. To make matters worse, evidence has accumulated to suggest that endothelial dysfunction is associated with an increased risk of T2DM through common pathological pathways described previously, synergistically resulting in a higher risk of diabetes-related vascular complications [67–69]. Given these pathophysiological associations, it is imperative to evaluate the therapeutic effect of glucose-lowering agents on endothelial dysfunction in patients with T2DM [30–32, 70]. The existing animal and human data suggest that SGLT2 inhibitors have favorable effects on several biomarkers related to arterial stiffness, myocardial workload, and SNS activity [47, 51, 71, 72], we seek to address whether empagliflozin modulates endothelial function dependently or independently of such markers as secondary endpoints in the trial proposed herein.

The assessment of endothelial function are clinically of importance to obtain the patients’ CV risk or prognosis, and to evaluate the effect of treatment [73, 74]. A number of reports show that T2DM was associated with abnormal endothelial function on the basis of various physiological functional tests, including RH-PAT [75–78]. Moreover, short-term treatment of some DPP-4 inhibitors improved RHI in patients with T2DM [30, 32], in contrast to large-scale outcome trials with DPP-4 inhibitors that failed to demonstrate superior CV safety compared with a placebo [38–40]. Although the reasons for this discrepancy are not yet fully understood, RH-PAT-guided assessments of endothelial function would make a promising tool for the evaluation of drug efficacy. Because treatment with SGLT2 inhibitors can favorably regulate hemodynamic aspects besides those influenced by glucose and lipid metabolism [13–17], it is highly likely that SGLT2 inhibitors impart a more direct and protective effect on the vascular system.

This trial has several limitations. First, it includes a relatively small sample size. Second, although the RH-PAT test is noninvasive, easy to perform, and an operator-independent method, its measurement is prone to be affected by individual physical conditions, intravascular volume, and the examination room environment. To minimize any such influences at participating institutions, investigators must perform the test in accordance with the EMBLEM-style operation manual which is modified from the manufacturer’s instruction (Additional file 4). In addition, because the empirical prediction implies that changes in the RHI in response to treatment may differ according to the RHI level, we plan to analyze data using subgroups stratified by the RHI level at baseline, as a subgroup analysis. Finally, SGLT2 inhibitors can potentially trigger some characteristic adverse drug reactions, such as intravascular volume depletion and urinary tract and genital infections, especially in the elderly and women [79]. Hence, investigators are cautioned to monitor patients for such reactions throughout the trial. In addition, information will be collected on predefined AESI as described earlier.

In summary, with the recent accumulation of evidence suggesting that SGLT2 inhibitors have CV and renal protective effects beyond glucose-lowering, the clinical use of SGLT2 inhibitors in patients with T2DM and established CVD could gain increased momentum.

Therefore, further assessments of the mechanisms that underpin the functionality of SGLT2 inhibitors are certainly warranted. The EMBLEM trial is the first study to assess the effect of empagliflozin on endothelial function in patients with T2DM and established CVD. In addition, possible mechanisms driving empagliflozin-mediated interactions between endothelial function and other CV markers will be evaluated. This trial has the potential to reveal profound mechanisms of action by which empagliflozin protects the CV system and improves CV outcomes in the EMPA-REG OUTCOME trial.

## Additional files

**Additional file 1.** Blood examination.

**Additional file 2.** AESI definitions.

**Additional file 3.** Trial organization.

**Additional file 4.** RH-PAT test manual.

## Abbreviations

AEs: adverse events
AESI: adverse events of special interest
AI: augmentation index
ANGPTL2: angiopoietin-like protein 2
baPWV: brachial–ankle pulse-wave velocity
BP: blood pressure
BW: body weight
CAD: coronary artery disease
CHF: chronic heart failure
CV: cardiovascular
CVD: cardiovascular disease
DSMB: Data and Safety Monitoring Board
HF: high frequency
HR: heart rate
HRV: heart rate variability
hs-TnI: high-sensitivity troponin-I
IL-8: interleukin-8
L-FABP: liver-type fatty acid-binding protein
LF: low frequency
LVEF: left ventricular ejection fraction
MMRM: mixed effects model for repeated measures
NT-proBNP: N-terminal pro-brain natriuretic peptide
NYHA: New York Heart Association
RAGE: receptor for advanced glycation end products
RHI: reactive hyperemia peripheral arterial tonometry index
RH-PAT: reactive hyperemia peripheral arterial tonometry
SGLT2: sodium glucose cotransporter 2
SD: standard deviation
SDNN: standard deviation of the normal to normal interval
SNS: sympathetic nervous system
T2DM: type 2 diabetes mellitus
