# Supplementary material for: Rationale and design of a multicenter placebo-controlled double-blind randomized trial to evaluate the effect of empagliflozin on endothelial function: the EMBLEM trial
Source: Cardiovasc Diabetol. 2017 Apr 12;16:48. doi: 10.1186/s12933-017-0532-8 (PMC5389095; doi:10.1186/s12933-017-0532-8)
Supplement: Supplementary file 2 — Additional file 2. AESI definitions. [file 12933_2017_532_MOESM2_ESM.docx]

**Additional File 2. AESI definitions**

**Adverse Events of Special Interest (AESI)**

The term AESI relates to any specific adverse event (AE) that has been identified at the project level as being of particular concern for prospective safety monitoring and safety assessment within this trial; for example, the potential for AEs based on knowledge derived from other compounds of the same drug class. AESIs are to be reported to the Pharmacovigilance Department of Boehringer Ingelheim within the same timeframe applicable to the reporting of serious AEs (SAEs).

Patients for whom an AESI is reported are to be followed up appropriately, regardless of the origin of the laboratory data (ie, central or local). The investigator should consider which, if any, concomitant therapies should not be administered during evaluation of the AESI. Discontinued treatments can be reintroduced at the discretion of the investigator.

The followings are considered an AESI:

Hepatic injury

A hepatic injury is defined by the following alterations of hepatic laboratory parameters after randomization:

- An elevation of aspartate aminotransferase (AST) and/or alanine aminotransferase (ALT) ≥ 3-fold upper limit of normal (ULN) combined with an elevation of total bilirubin ≥ 2-fold ULN measured in the same blood sample
- An isolated elevation of ALT and/or AST ≥ 5-fold ULN

These laboratory findings constitute a hepatic injury alert and the patients with these abnormalities need to be followed up according to medical judgement.

In the case of clinical symptoms of hepatic injury (eg, icterus, unexplained encephalopathy, unexplained coagulopathy, right upper quadrant abdominal pain, etc.) in the absence of laboratory results (ie, ALT, AST, and total bilirubin), the investigator is required to confirm these parameters are analysed, if necessary in an unscheduled blood test.

Decreased renal function

Decreased renal function is defined as a creatinine value with a ≥ 2-fold increase from baseline and above the ULN.

For the AESI “decreased renal function,” the investigator shall collect an unscheduled laboratory sample for creatinine measurement as soon as possible and initiate follow-up laboratory creatinine tests according to medical judgement.

Metabolic acidosis, ketoacidosis and diabetic ketoacidosis (DKA)

In the case of metabolic acidosis, ketoacidosis, and DKA, further investigations are required according to medical judgment and the clinical course of the complication until a diagnosis is made and/or the patient has recovered.

DKA is defined by the American Diabetes Association (ADA) diagnostic criteria as outlined in the table below.

Investigators are required to note that not all criteria in the table below need to apply for the diagnosis of DKA, and clinical judgment should also be taken into consideration. Due to its mechanism of action, empagliflozin may potentially modify the clinical presentation of DKA which may occur at lower plasma glucose levels than are stated in the table below.

**Table.** Diagnostic criteria for DKA

|  | DKA | | |
| --- | --- | --- | --- |
|  | Mild | Moderate | Severe |
| Plasma glucose (mg/dL) | > 250 | > 250 | > 250 |
| Arterial pH | 7.25-7.30 | 7.00-7.24 | < 7.00 |
| Serum bicarbonate (mEq/L) | 15-18 | 10 to < 15 | < 10 |
| Urine ketones* | Positive | Positive | Positive |
| Serum ketones* | Positive | Positive | Positive |
| Effective serum osmolality (mOsm/kg)** | Variable | Variable | Variable |
| Anion gap*** | > 10 | > 12 | > 12 |
| Alteration in sensoria or mental obtundation | Alert | Alert/drowsy | Stupor/coma |

*Nitroprusside reaction method.

**Calculation: 2 [measured Na (mEq/L) + glucose (mg/dL)]/18.

***Calculation: (Na^+^) – (Cl^-^ + HCO_3_^-^) (mEq/L).

Events involving lower limb amputation

This definition includes amputation (ie, resection of a limb through a bone), disarticulation (ie, resection of a limb through a joint), and auto-amputations (ie, spontaneous separation of the nonviable portion of the lower limb).

Not included in this definition are debridement (ie, removal of callused or dead tissue), procedures on a stump (such as stump revision, drainage of an abscess, wound revision, etc.) and other procedures (eg, nail resection or removal) without the concomitant resection of a limb (ie, amputation or disarticulation).

Each lower limb amputation, disarticulation, or auto-amputation is to be reported separately. The SAE report is to include the date of the procedure, the level of amputation or disarticulation, the medical condition(s) leading to the procedure, and if the patient had some known risk factor(s) for lower limb amputation.
