# Supplementary material for: Rationale and design of a multicenter placebo-controlled double-blind randomized trial to evaluate the effect of empagliflozin on endothelial function: the EMBLEM trial
Source: Cardiovasc Diabetol. 2017 Apr 12;16:48. doi: 10.1186/s12933-017-0532-8 (PMC5389095; doi:10.1186/s12933-017-0532-8)
Supplement: Supplementary file 4 — Additional file 4. RH-PAT test manual. [file 12933_2017_532_MOESM4_ESM.docx]

**Additional File 4. RH-PAT Test Manual**

# **Basic instructions**

As a rule, this test will be carried out as described below.

Measurements will be taken in the morning (before 11 AM), and as a rule, both the baseline and 6-month measurements will be taken at the same time of day.

# **Advance information for participants**

[1] Subjects may not eat breakfast on test days (Avoid eating any food after 22:00 on the day before the test)

Water alone may be ingested. However, after arriving at the hospital until immediately before the test, only water at room temperature is permitted.

(Be sure to hydrate as needed until just before the test, especially during the summer to avoid dehydration).

Drinking tea, coffee, milk, or juices, including sugar intake such as candies, is prohibited until the test is complete.

[2] Do not take any medications on the day of testing (Only take the medications after measurements are completed).

[3] Those who regularly take nitroglycerine formulations should stop taking nitroglycerine at least 24 hours before the test. This also applies to nitrate patches which should not be applied after bathing on the day before the test. (This washout period may be altered depending on the physician’s discretion)

[4] Morning baths and exercise such as jogging is prohibited on the day of the test.

[5] Subjects taking ED drugs will be required to discontinue use 24 hours before the test regardless of the drug’s duration of action.

[6] Smokers are asked to avoid smoking from 3 hours before the test.

[7] Please use the toilet before the test (You will not be able to go during the test).

[8] If blood is drawn on the day of the test, samples will be taken from your dominant side.

# **Test room conditions**

[1] The room must be quiet.

1) Outside noises should not be audible (Use of ear plugs is permitted)

2) Avoid allowing entry and exit of staff members

[2] Adjust light sources so the light does not strike the subject’s eyes (administer test under dimmed light conditions)

[3] Ensure that the subject feels neither too hot nor too cold by maintaining a comfortable room temperature

(Recommended range: 21 to 26ºC) with an air conditioner.

# **Measurement procedures**

[1] Device preparation

1. Turn on the system and allow it to warm up for 20 minutes before starting measurements. (Do not turn off the device until the final measurement of the day is completed)

[2] Patient preparation

1) Have subjects change into clothing appropriate for measurement (occlusion)* and lie down and relax
in the bed provided for 15 to 20 minutes. (In the winter, a 30-minute rest period is recommended to allow fingertip vasoconstriction to normalize).

*There are no specific clothing restrictions so long as it does not hamper occlusion. During the winter, avoid heavy sweaters or other thick clothing. Instead, wear long-sleeved shirts and other lightweight clothing.

2) Have subjects remove any clothing that might restrict blood flow to the arm.

3) Have subjects remove any accessories such as rings, jewelry, watches, bracelets, or glasses.

4) Turn off all mobile phones or other electronic devices and place them at least 2 meters away from the subject.

5) Measure blood pressure on the dominant arm (control arm) and record both systolic and diastolic blood pressures in a supine position. (Leave at least 5 minutes between blood pressure measurement and test measurements)

6) Cover the subject with a towel or blanket to relax the subject.

7) Although any digit other than the thumb can be used as the test finger, measurements are generally taken with the second finger. (Use the same finger on both sides)
8) Cut nails if they are too long (Nails may damage the rubber inside the probe, or may displace fingertips in the sensor area)
9) Explain that measurements will be taken 6 minutes before occlusion, after 5 minutes of occlusion, and 5 minutes after release.

10) Explain that although the upper arm will be constricted with a blood pressure cuff during measurement, there is nothing to worry about. (Explain that it is important to bear the discomfort of occlusion to take accurate measurements.)
11) During the test, the patient must not move (be especially careful not to move the arms). Check to see that the patient is comfortable in this position.

12) Explain that the patient is free to fall asleep.

[3] Measurement

1) Take measurements at: 6 minutes before occlusion (baseline)
 　　　5 minutes during occlusion (confirm complete occlusion)

5 minutes after release

1. Place a blood pressure (BP) cuff on the upper arm of the non-dominant arm (contralateral from arm used for BP measurement).
2. Inflate the BP cuff to either 60 mmHg above systolic pressure or 200mmHg, whichever is higher.
3. If occlusion is insufficient, continue in inflate in 50mmHg increments, but do not exceed 300mmHg
4. To check for occlusion, see if pulse waveforms on the control arm and occluded arm are asynchronous (To check for synchronicity, set time scale at 15 seconds and signal gain on occluded side at 20,000)

# **Miscellaneous** [1] Be sure to come in early when you visit the hospital for measurements.

This is particularly important during the cold season, since you will need some time to rest and relax before the test. The “original endothelial function status” can be observed by minimizing any sympathetic nerve influences.

# **Export Data**

Export the following items into PDF format and upload to EDC.

| Item |  |  |
| --- | --- | --- |
| Test date |  |  |
| ID | Depends on data input at each institution |  |
| Age |  |  |
| Sex |  |  |
| Height |  |  |
| Weight |  |  |
| BMI |  |  |
| Blood pressure (systolic) |  |  |
| Blood pressure (diastolic) |  |  |
| Note 1 |  |  |
| Note 2 |  |  |
| Comment |  |  |
| Measured time | Total measured time |  |
| Measured by |  |  |
| Measurement version | Software version |  |
| Analysis version | Software version |  |
| Occlusion time | Generally, the display should say, "Automated" |  |
| Baseline | Resting state measurement |  |
| Occlusion | Occlusion time |  |
| Dilatation | Resting time after release |  |
| RHI | Reactive Hyperemia Index |  |
| Heart Rate |  |  |
| AI | Augmentation Index | AI-related |
| AI%75bpm | AI with Heartrate adjusted to 75 beats |  |
| Mean | Mean pulse during AI calculation |  |
| Mean NN | Mean RR interval | HRV-related |
| SDNN | Standard deviation of the normal to normal intervals |  |
| RMSSD | The square root of the mean of the squares of the successive differences between adjacent RRs |  |
| NN50 | The number of pairs of successive RRs that differ by more than 50 ms |  |
| pNN50 | The proportion of RR50 divided by total number of RRs |  |
| Triangular Index |  |  |
| LF | Low frequency |  |
| HF | High frequency |  |
| LF/HF | Power ratio of low and high frequencies |  |
